# Learning on the job? Foraging strategies of juvenile versus adult Lesser black-backed gulls at their first migratory stopover

**DOI:** 10.1098/rsos.241224

**Published:** 2024-12-11

**Authors:** Mélibée Morel, Reinoud Allaert, Eric Stienen, Ruben Fijn, Frederick Verbruggen, Wendt Müller, Luc Lens

**Affiliations:** ^1^ Department of Biology, University of Antwerp, Behavioural Ecology and Ecophysiology Group, Campus Drie Eiken Universiteitsplein 1, 2610 Wilrijk, Antwerp, Belgium; ^2^ Department of Biology, Ghent University, K. L. Ledeganckstraat 35, 9000 Ghent, Belgium; ^3^ Ghent University, Centre for Research on Ecology, Cognition and Behaviour of Birds, 9000 Ghent, Belgium; ^4^ Department of Experimental Psychology, Ghent University, Henri Dunantlaan 2, 9000 Ghent, Belgium; ^5^ Research Institute for Nature and Forest (INBO), Herman Teirlinckgebouw, Havenlaan 88, 1000 Brussels, Belgium; ^6^ Waardenburg Ecology, Varkensmarkt 9, 4101 CK Culemborg, The Netherlands

**Keywords:** early development, age-dependent foraging strategies, lesser black-backed gull, migratory stopover, learning, GPS tracking

## Abstract

Developing efficient foraging strategies is critical for survival, especially during the high-mortality post-fledging period in birds. This period is particularly challenging for migratory species, where juveniles must navigate unfamiliar environments with limited experience and knowledge. Our study focused on the foraging strategies of 20 juvenile lesser black-backed gulls (*Larus fuscus*) during the first 20 days of their initial migratory stopover. We assessed learning through changes in their spatial (re)use and activity patterns using GPS tracking data, in direct comparison with similar data collected from 38 experienced adults. Juveniles were less exploratory and spent more time foraging than adults, but showed similar spatial consistency. Over time, both juveniles and adults reduced their range distribution areas, but only adults significantly reduced their flying time. Adults exhibited space use optimization by travelling shorter distances and spending progressively more time foraging. In contrast, juveniles showed no clear evidence of spatial learning or improved foraging skills, as there was no decrease in cumulative distance travelled nor a clear pattern in time spent foraging.

## 1. Introduction

There is considerable evidence that early life stages represent a pivotal period for the development of many phenotypic traits, with potentially lasting consequences [1,2]. During this time, individuals undergo a critical transition to independence that can shape their future survival and reproductive success. In avian species, the post-fledging stage is critical as it acts as a selection filter due to its inherently high mortality rates (e.g. [3,4]). Among others, it has been hypothesized that factors such as physical immaturity and inexperience represent important determinants of the survival probability during this vulnerable period [5–8]. Quickly gaining experience and acquiring the relevant skills are therefore crucial. However, the mechanisms by which juveniles develop essential life skills, such as effective foraging strategies, remain poorly understood.

Three factors stand out to describe how crucial the juvenile phase is. First, when young individuals must fend for themselves for the first time, they are often navigating unfamiliar environments. Thus, juveniles are expected to behave in a more exploratory way, as their lack of spatial knowledge can lead to reduced efficiency in locating profitable foraging sites [9,10]. While this might imply that juveniles must travel longer distances to meet their daily energy needs, they face an additional challenge due to their physical immaturity and poor flight skills [11–13], both of which likely increase flight costs. Second, juveniles lack experience in foraging, showing more limited food acquisition skills, as reflected in their lower foraging success [14], so they might have to invest more time and effort into foraging [3,15–18]. Third, juveniles are often outcompeted by more experienced birds [19,20] and may lose food to others through kleptoparasitism [21,22]. Juveniles may thus have to invest more time in foraging to compensate for their inferior competitiveness. Together, these factors challenge the critical goal of achieving a positive energy balance during this formative period of their life cycle, and hence their ability to survive.

The rapid acquisition of experience and the development of the ability to navigate, locate, and exploit foraging skills are crucial for juveniles. Previous studies have highlighted the importance of individual exploration, during which juveniles use landscape features to form spatial memories that allow them to identify and return to profitable foraging sites [9,10,16,23,24]. This process of exploration and memory formation is critical for the development of efficient foraging. Furthermore, trial-and-error learning can play a role in the refinement of foraging skills over time [25,26]. Social interactions with conspecifics might reinforce both learning processes [27], providing valuable opportunities for young animals to learn about foraging sites and further develop their foraging strategies [28–30].

Using GPS tracking, we analyse here how juvenile lesser black-backed gulls (*Larus fuscus*) navigate new environments during their first migration stopover and assess their capacity for improvement and learning by comparing their spatial use and activity pattern over time to that of adults. We selected this species because previous long-term tracking studies have provided important insights into the spatial use and foraging strategies of adult lesser black-backed gulls, leading to a fundamental understanding of their ecology [31–34]. Furthermore, due to the migratory nature of lesser black-backed gulls, a comparative analysis between juveniles and adults during the post-fledging migratory phase offers a unique perspective. Juveniles lack prior experience with the migratory stopover habitats, unlike adults who are likely to be familiar with these sites or, at least, similar sites through previous migratory cycles [35,36]. Thus, we use adults as a baseline in this comparative study. We hypothesized that juveniles, lacking spatial familiarity, would initially exhibit more extensive exploratory behaviours (e.g. roaming larger areas and travelling longer distances) and less consistency in their spatial patterns (e.g. less spatial reuse) than adults. In addition, we expected that juveniles, being less skilled at foraging, would devote more time to active foraging and consequently reduce their resting time compared to adults. Through the process of learning, including spatial learning (broadly defined; e.g. finding profitable sites, improving searching abilities and timely switching to new foraging sites when one is depleted), as well as behavioural learning (e.g. reducing handling times, improving food recognition), we hypothesized that the initial differences in foraging behaviour between juveniles and adults would diminish over time.

## 2. Material and methods

### 2.1. Study area and GPS tracking

A total of 87 juvenile lesser black-backed gulls (45 individuals in 2020 and 42 individuals in 2021) from the Zeebrugge harbour colony (Belgium, 51°20′ N, 3°10′ E) and 75 breeding adult lesser black-backed gulls (25 individuals in 2020 and 50 individuals in 2021) from the Neeltje Jans colony (The Netherlands, 51.62° N, 3.68° E) were fitted with solar-powered Ornitela OT-15-3G GPS devices. The two sites, 47 km apart, are characterized by artificial, sparsely vegetated and sandy environments, with the Neeltje Jans colony containing approximately 3000 breeding pairs and the Zeebrugge colony approximately 300 breeding pairs.

GPS devices (including Teflon ribbon wing harnesses) weighed approximately 2.5% of the bird’s body mass [37,38] and were configured with a minimum resolution of 20 min, with most devices set to record location data at this interval, while others were configured to record data at a higher frequency. Adult gulls were captured directly on their nests during the breeding season using walk-in traps on specific dates (20 and 28 May 2020; 25 May, 1 and 2 June 2021). In contrast, juvenile gulls in the Zeebrugge colony were captured by hand at their nest at 30 days of age, when their flying ability was still limited. Juveniles were sexed via PCR using feathers collected at hatching, following Fridolfsson & Ellegren [39], with the CHD1W and CHD1Z introns targeted using 2550F/2718R primers. PCR conditions involved 30 cycles with an annealing temperature of 56°C. Adults were sexed using morphometric measurements (weight, wing and head length). Due to the risk of predation by local foxes, which resulted in the immediate loss of at least 10 tagged chicks in 2020, in 2021 the juveniles were temporarily transferred to a secure aviary in Ostend for a further five weeks, after which they were tagged and released on a protected estuarine beach about 30 km from their original colony.

### 2.2. Data processing

The GPS data were pre-processed in R version 4.3.1 [40]. First, we enriched the GPS data with land-use information sourced from the Moderate Resolution Imaging Spectroradiometer (MODIS 500 m type1) available in the NASA database [41]. These data were categorized into three habitat types: agricultural, marine and urban. Agricultural habitats included ‘savannas’ (i.e. tree cover 10–30%), ‘grasslands’ (i.e. dominated by herbaceous annuals (<2 m)), ‘croplands’ (i.e. at least 60% of area is a cultivated cropland) and ‘cropland/natural vegetation mosaics’ (i.e. mosaics of small-cultivation 40–60% with natural tree, shrub or herbaceous vegetation). Marine habitat corresponded to ‘water bodies’ (i.e. at least 60% of area covered by permanent water bodies). Urban habitat corresponded to ‘urban/built-up lands’ (i.e. at least 30% impervious surface area including building materials, asphalt and vehicles).

Behavioural categories (‘foraging’, ‘flying’ or ‘resting’) were assigned to each GPS fix using a random forest classifier, trained by Kavelaars *et al.* [42]. The model used a combination of parameters, including ground speed, turning angle, step length and MODIS land-use data. The classifier was trained and validated on expert-annotated data and optimized using multiple input streams, achieving high accuracy (83.2%) in distinguishing behaviours [42].

We only retained GPS fixes recorded after the onset of migration, which we defined as the first instance when a bird moved more than 5 km away from the starting location and did not return within the same year [43,44]. For birds that began migration from the colony, the starting location was the colony itself. However, for the 2021 juveniles that were released 30 km from the colony, we used the release site as the starting location. If necessary, the data were subsampled to a resolution of 20 min to avoid any sampling bias.

**Figure 1.**
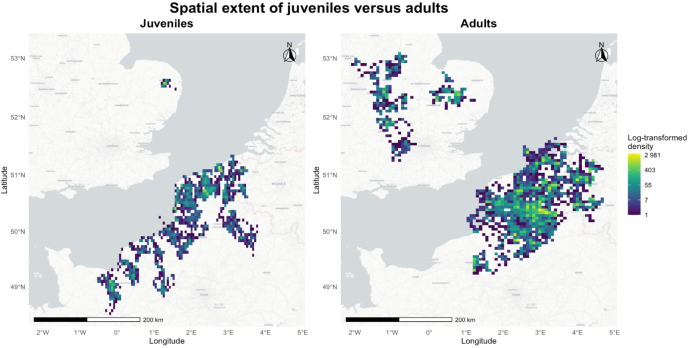
Spatial extent of first stopover sites for juvenile and adult lesser black-backed gulls. The map shows the log-transformed density of GPS locations for juveniles (left) and adults (right) during their first migratory stopover.

To identify stopovers during migration, we applied density-based spatial clustering to the migratory GPS data of 72 adults and 51 juveniles that successfully initiated migrating, using a noise algorithm as implemented in the ‘dbscan’ package (version 1.1-11) [45]. Note that initial sample sizes were higher, but some animals died before migration began. Non-clustered data were considered as migratory bouts [35], while the clusters represented ‘stopovers’ (i.e non-migratory bouts). The neighbourhood radius was set to 0.2° (i.e approximately 18 km) to distinguish between migratory movements and foraging movements during stopovers. The spatial extent of these first stopover sites is illustrated in figure 1, showing the log-transformed density of GPS locations for juveniles and adults. The optimal epsilon value for the neighbourhood radius was determined visually using the ‘kNNdistplot’ function from the ‘dbscan’ package. Clusters were defined by a minimum of 144 points, corresponding to a minimum duration of two days at a 20 min resolution. We only analysed the data of individuals on their first stopover, and only retained those individuals that stayed there for at least 20 days. This criterion allowed us to focus on individuals with a more stationary migratory strategy, providing an opportunity to observe potential learning behaviours in a consistent environment over an extended time frame.

This stopover and time frame were chosen to maintain a sufficiently large sample size and because we expect that the strongest learning effects in a new environment will occur specifically during this first stopover, in the initial stages of exploration. Using this criterion, we retained GPS data of 45 adults and 33 juveniles, allowing us to examine temporal variation in spatial use and foraging behaviour. We excluded one adult and one juvenile with data gaps exceeding two hours (electronic supplementary material, figure S1). Additionally, to avoid the influence of birds that might have been in poor body condition and potentially exhibited abnormal behaviour, we excluded eleven juveniles and three adults that died within 14 days after leaving the stopover. We also removed two individuals from the study due to suboptimal device performance, resulting in fewer than 1000 fixes (equivalent to a resolution of approximately 30 min or less). We divided the 20 days at the stopover into four equal periods of five days each, which we considered as the minimum number of GPS fixes required to reliably calculate spatial use parameters. Based on the coordinates and visual inspection of the stopovers, we excluded one individual whose first stopover was at a much more distant location than the others. This ensured behavioural comparisons were made within similar ecological contexts. In the end, 20 juveniles (11 females and 9 males) and 38 adults (19 females and 19 males) were included in the analysis (electronic supplementary material, table S3).

**Figure 2.**
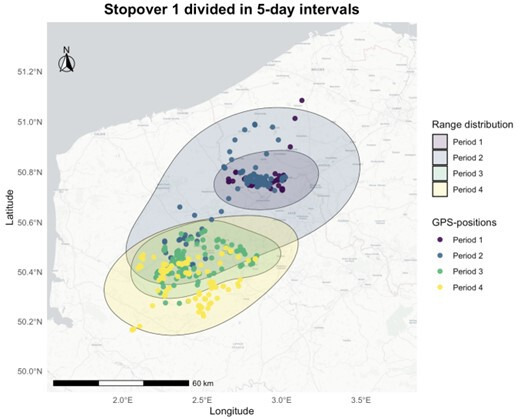
Illustration of range distribution areas and their overlap during an individual’s first migratory stopover, used to assess spatial (re)use.

To assess spatial use and reuse as well as temporal variability, we used autocorrelated kernel density estimation (AKDE; 95% home range area) to estimate range distribution areas [46] and their overlap over consecutive time periods (figure 2). Range distribution areas, here defined as the 95% contour of its utilization distribution, represent the spatial extent over which an animal moves and conducts its local activities. The AKDE is particularly suited for studying movement behaviours as it accounts for the autocorrelated nature of the data, thereby reducing biases associated with inconsistent tracking time lags [47,48]. We calculated home ranges using the ‘hr_akde’ function from the ‘amt’ package (version 0.2.1.0) [49]. As noted previously, prior to the AKDE calculations, ‘stopovers’ were identified as clustered, non-migratory bouts using density-based spatial clustering, which confirmed range residency during periods when individuals were stationary, allowing accurate application of AKDE to these specific intervals. Movement models, including Ornstein–Uhlenbeck (OU), Ornstein–Uhlenbeck foraging (OUF) and independent identically distributed (IID) processes, were fitted to the data using the ‘fit_ctmm’ function within the AKDE calculations. The model fitting method was automatically selected to best describe the movement process. Models were selected based on the lowest delta Akaike information criterion corrected (dAICc) values. In addition, range overlaps between consecutive periods were calculated using the ‘hr_overlap’ function from the ‘amt’ package utilizing Bhattacharyya’s affinity to measure the degree of overlap [50].

To assess daily movement patterns and foraging effort, we calculated the cumulative distance by summing the Haversine distances between consecutive GPS positions for each period. Then, we calculated the scaled cumulative distance by normalizing the cumulative distance divided by the number of GPS fixes in the same period to reduce any sampling bias. In addition, we quantified the relative frequency of the classified behaviours—flying, foraging and resting—as measures of foraging effort and foraging efficiency by calculating the ratio of GPS fixes annotated with each behaviour to the total number of GPS fixes during the day. Contrary to the home range analysis, this analysis was restricted to daylight hours only, as individuals are mostly inactive at night [35].

### 2.3. Data analysis

Prior to the analyses, we investigated whether colony size influenced the exploratory behaviour of birds, which could manifest in migratory and foraging strategy differences. We analysed GPS data from adult gulls in both colonies across 2020 and 2021. Using a linear model (LM) with a Gaussian distribution, we assessed cumulative distances travelled, incorporating sex, year and colony as explanatory variables. The analysis showed no significant effect of colony on travel distances (electronic supplementary material, table S1), suggesting that colony in this study does not influence exploratory behaviour. Additionally, we analysed to assess whether captivity influenced spatial use, foraging effort or efficiency of juveniles, given the potential impact of the captivity period on their foraging behaviour. The results indicated no significant effect of the year (electronic supplementary material, table S2), suggesting that captivity did not measurably affect the foraging behaviour of juveniles during their first stopover. Alongside the formal model, we also examined the raw data and found no substantial numerical differences between years.

Both juvenile and adult lesser black-backed gulls predominantly used agricultural fields during their first stopover (mean of 76% for adults and 87% for juveniles; electronic supplementary material, figure S2), and the species is known to have different activity patterns depending on the habitat type (e.g. longer but less energy-demanding terrestrial foraging trips compared to marine trips) [33]. Therefore, we first investigated whether the proportion of agricultural habitat use per period differed between life stages and over time. The proportion of agricultural habitat use was quantified as the ratio of GPS fixes within agricultural habitats to the total number of GPS fixes collected during daylight hours. To test this, we used a generalized linear mixed model (GLMM) with a beta distribution, implemented in Template Model Builder (‘glmmTMB’). The model included the following predictors: life stage, period, the interaction between life stage and period, sex, year of tagging and the total duration of the stopover measured in days (i.e. the actual time spent at the first stopover). Individual ID was included as a random effect. As this analysis revealed significant effects of period and life stage on the proportional use of agricultural habitat (electronic supplementary material, table S4), both the proportion of agricultural habitat used and its interactions with period and life stage were included in all subsequent models.

Following our preliminary analysis, we examined the effects of life stage and period on spatial use. To this end, we fitted a linear mixed model (LMM) with a Gaussian distribution to analyse the log-transformed range distribution areas, ensuring the normality of the data. We then estimated the influence of life stage and period on spatial reuse. To analyse these proportions of overlap, we fitted a GLMM with a beta distribution using a ‘glmmTMB’, which allowed us to understand potential changes in range distribution over time.

Next, to assess the effects of life stage and period on foraging effort, we used an LMM with a Gaussian distribution to analyse scaled cumulative distances travelled per period. This scaling was relative to the number of GPS positions collected per period. Finally, we estimated the impact of life stage and period on the foraging effort and efficiency by fitting several LMMs with Gaussian distributions to the relative proportions of foraging, flying and resting behaviours, thereby assessing the time allocation between different behavioural activities.

All models included the variables life stage and period, along with their interaction. Additional covariates in all models included sex, year of tagging, total duration of the stopover and proportion of agricultural habitat use (over the 20 days for the overlap model and per period for the other models), along with their interactions with period and life stage. The total duration of stopover and the proportion of agricultural habitat use were scaled and centred using the scale() function. To account for potential curvilinear relationships, quadratic effects of these covariates were integrated to improve model fit. However, in models where these quadratic effects were found to be non-significant, they were subsequently removed to avoid overfitting and to simplify interpretation. Bird ID was included as a random intercept to control for pseudo-replications associated with repeated measures from the same individuals. Non-significant interactions were systematically removed and the results of these reduced models are reported in table 1. We used the ‘lme4’ (version 1.1-34) [51], ‘lmerTest’ (version 3.1-3) [52] and ‘glmmTMB’ (version 1.1.7) [53] packages in R for model fitting. Statistical significance was maintained at an alpha level of 0.05. Model fits and residuals were evaluated using the ‘performance’ (version 0.10.4) [54] and ‘DHARMa’ (version 0.4.6) [55] packages. Data visualization was conducted using ‘ggplot2’ (version 3.4.3) [56].

**Table 1.** Results of regression analyses testing whether adults and juveniles differ in spatial use (1 and 2), foraging effort and efficiency (3, 4a–c), and whether these differences change over time. The reduced models are reported in this table, with juveniles as the baseline group in period 1 (i.e. the negative estimates for life stage in the models indicate that juvenile parameters are lower than those of adults). Full models are presented in the Additional file 2. Covariates are shown in *italics*, significant relationships are shown in **bold**.

	estimate ± s.e.	df	*p* value
1. Range distribution areas (km^2^)			
**Intercept**	**9.24 ± 0.53**	60	**<0.001**
**Period**	**−0.44 ± 0.07**	175	**<0.001**
**Life stage**	**−1.24 ± 0.44**	53	**0.007**
*Sex*	−0.58 ± 0.41	52	0.16
*Total duration of stopover*	−0.16 ± 0.21	50	0.45
*Year of tagging*	−0.30 ± 0.40	50	0.46
***Proportion of agricultural habitat use***	**−0.66 ± 0.24**	213	**0.006**
***Life stage × proportion of agricultural habitat use***	**0.77 ± 0.33**	155	**0.02**
*(Total duration of stopover)^2*	−0.16 ± 0.19	51	0.39
***(Proportion of agricultural habitat use)^2***	**−0.35 ± 0.09**	222	**<0.001**
2. Range overlap (proportion)			
Intercept	0.32 ± 0.34		0.35
Period	0.03 ± 0.08		0.68
Life stage	0.37 ± 0.23		0.1
*Sex*	0.07 ± 0.22		0.75
*Total duration of stopover*	−0.009 ± 0.11		0.94
*Proportion of agricultural habitat use*	−0.11 ± 0.11		0.30
*Year of tagging*	0.38 ± 0.21		0.07
3. Scaled cumulative distance travelled			
Intercept	0.11 ± 0.12	77	0.37
**Period**	−0.11 ± 0.02	174	**<0.001**
**Life stage**	−0.65 ± 0.14	135	**<0.001**
*Sex*	−0.12 ± 0.11	53	0.27
*Total duration of stopover*	−0.08 ± 0.05	52	0.13
*Year of tagging*	0.12 ± 0.10	51	0.24
***Proportion of agricultural habitat use***	0.08 ± 0.04	172	**0.04**
**Periods × life stage**	0.08 ± 0.04	170	**0.02**
4a. Time spent flying (proportion)			
**Intercept**	0.17 ± 0.02	65	**<0.001**
**Period**	−0.009 ± 0.002	176	**<0.001**
Life stage	0.005 ± 0.01	52	0.73
*Sex*	−0.02 ± 0.01	51	0.11
*Total duration of stopover*	−0.01 ± 0.006	50	0.08
*Year of tagging*	−0.006 ± 0.01	49	0.64
*Proportion of agricultural habitat use*	−0.01 ± 0.007	214	0.15
*(Total duration of stopover)^2*	−0.002 ± 0.006	51	0.75
***(Proportion of agricultural habitat use)^2***	−0.009 ± 0.003	222	**0.007**
4b. Time spent foraging (proportion)			
**Intercept**	0.60 ± 0.02	81	**<0.001**
**Period**	0.01 ± 0.003	172	**<0.001**
**Life stage**	0.07 ± 0.02	166	**<0.001**
Sex	0.02 ± 0.01	51	0.11
Year of tagging	−0.007 ± 0.01	49	0.54
***Proportion of agricultural habitat use***	0.18 ± 0.009	221	**<0.001**
*Total duration of stopover*	0.009 ± 0.006	50	0.14
**Periods × life stage**	−0.02 ± 0.005	168	**0.002**
***Periods × proportion of agricultural habitat use***	0.005 ± 0.002	175	**0.03**
***(Proportion of agricultural habitat use)^2***	0.007 ± 0.003	214	**0.02**
*(Total duration of stopover)^2*	−0.006 ± 0.005	50	0.26
4c. Time spent resting (proportion)			
**Intercept**	−1.71 ± 0.09	63	**<0.001**
Period	0.008 ± 0.01	176	0.52
Life stage	−0.09 ± 0.08	54	0.26
Sex	0.003 ± 0.07	52	0.97
Total duration of stopover	0.05 ± 0.04	50	0.14
Year of tagging	0.02 ± 0.07	50	0.77
**Proportion of agricultural habitat use**	−0.96 ± 0.04	202	**<0.001**
***Life stage × proportion of agricultural habitat use***	−0.25 ± 0.06	138	**<0.001**
*(Total duration of stopover)^2*	0.06 ± 0.03	52	0.07
***(Proportion of agricultural habitat use)^2***	−0.19 ± 0.02	220	**<0.001**

## 3. Results

### 3.1. Spatial (re)use

**Figure 3.**
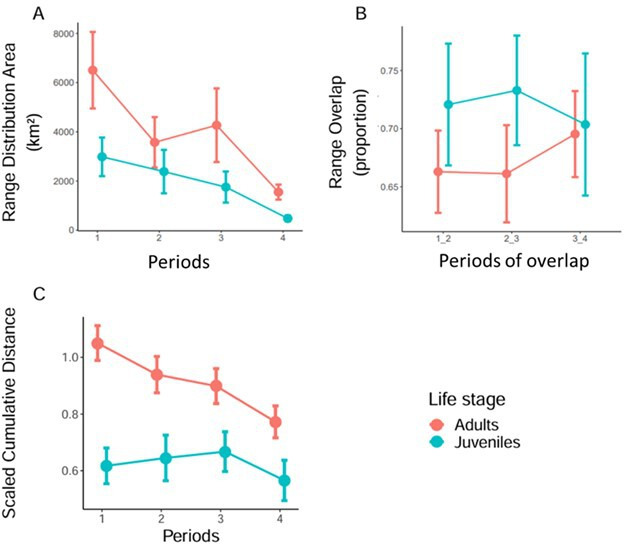
Ranging behaviour of juvenile and adult lesser black-backed gulls over five-day periods during their first migratory stopover. Bars are standard errors of the mean. (*a*) Mean size of range distribution areas. (*b*) Mean proportion of overlap of range distribution areas. Periods of overlap ‘*x*_*y*’ indicate the proportion of overlap of the area from period *y* with the area from period *x*. (*c*) Mean scaled cumulative distance travelled in kilometres between two fixes.

Juveniles occupied smaller range distribution areas than adults, with both life stages showing a decrease in spatial use over time (table 1, figure 3*a*). In addition, no significant effects of life stage or period were found on the proportion of range overlap between consecutive periods, and no statistically significant temporal change was observed (table 1, figure 3*b*).

### 3.2. Foraging

For scaled cumulative distance travelled, there was a significant interaction effect between life stage and period (table 1). Juveniles travelled less than adults, but this difference decreased over time as juvenile distances travelled remained fairly constant (Estimate = −0.03, s.e. = 0.03, *p* = 0.28; electronic supplementary material, table S5), while adults travelled less (Estimate = −0.12, s.e. = 0.02, *p *< 0.001; figure *3c*).

**Figure 4.**
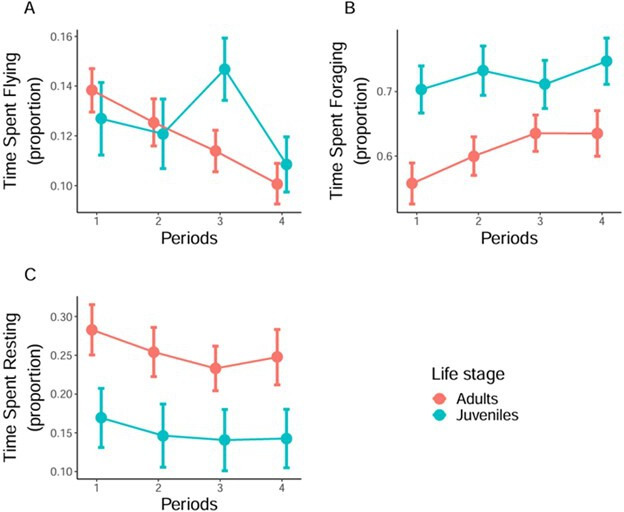
Flight, foraging and resting behaviour of juvenile and adult lesser black-backed gulls over five-day periods during their first migratory stopover. Bars are standard errors of the mean. (*a*) Mean relative proportion of time spent flying. (*b*) Mean relative proportion of time spent foraging. (*c*) Mean relative proportion of time spent resting.

There was no significant effect of life stage on the relative proportion of flying (table 1). However, both juveniles and adults flew proportionally less over time as indicated by the significant negative effect of period (table 1, figure 4*a*). The relative proportion of foraging was higher for juveniles than for adults (table 1). Yet in adults, but not in juveniles, the relative proportion of foraging increased over time (Estimate = 0.01, s.e. = 0.003, *p *< 0.001 for adults; Estimate = −0.006, s.e. = 0.004, *p* = 0.21 for juveniles; figure 4*b*, electronic supplementary material, table S6). Finally, there was no significant overall difference in resting time between juveniles and adults, nor was there any temporal variation (table 1).

## 4. Discussion

The main objective of this study was to gain insight into the early development of foraging strategies in naive juvenile lesser black-backed gulls by quantifying temporal changes in spatial (re)use, cumulative travel distance and time spent flying, foraging and resting at their first migratory stopover site and to compare their activity patterns with that of experienced adults, used as a baseline for this study. We hypothesized that juveniles, due to lack of spatial knowledge, would initially explore more while showing less consistent spatial patterns than adults. Furthermore, given their limited foraging skills, they might have to spend more time foraging and less time resting. These differences were expected to diminish as juveniles would learn and gain experience. Contrary to our expectations, juveniles roamed smaller areas, did not show a different degree of consistency in spatial use and travelled shorter distances than adults. Even so, as expected, they initially spent more time foraging than adults. Over time, both juveniles and adults roamed smaller areas, but only adults showed a reduction in flying time. The anticipated gradual decrease in distance travelled was observed exclusively in adults, who also progressively spent a greater proportion of their time foraging.

The observation that juveniles initially roamed smaller areas and travelled shorter distances may be due to poorer flight skills associated with their physical immaturity [11,12], particularly because shorter wing length can constrain flight abilities and increase flight costs overall [57,58]. Developing these flight skills, such as efficient wind use and soaring flight modes, can take several months [13,59], requiring juveniles to learn the finer aspects of flying, including the use of orographic lifts [60,61]. Alternatively, or in addition, it may reflect their unfamiliarity with the novel environment. A lack of prior spatial knowledge, including information on foraging patch quality, may lead juveniles to spend more time at foraging sites, even when they are not profitable [62], impacting their decision-making when switching to nearby locations. We found no effect of life stage on the range overlap between consecutive periods, suggesting that juveniles revisited known foraging sites as often as adults. For naive birds, this may indicate regular updating of information to compensate for their inexperience and improve their understanding of resource availability [63], as well as a higher reliance on sites where they have previously found resources [62], rather than exploring new and potentially better sites. However, this study focused on birds that remained at the stopover for at least 20 days, allowing us to examine learning processes in a stable environment. While this approach provided valuable insights into the spatial behaviour of individuals with a more stationary strategy, it excluded birds with shorter stopovers or more continuous migratory movements. These birds may exhibit different learning capacities, which future research could explore.

Although we lack direct observations of foraging efficiency, the fact that juveniles initially spent more time foraging than adults aligns with previous studies indicating lower foraging efficiency in younger individuals [3,15,16,64]. In many species, young and inexperienced individuals face high mortality due to poor feeding techniques and inadequate anti-predatory behaviour. For example, independent juvenile European shags (*Phalacrocorax aristotelis*) spend up to twice as much time foraging as adults, compensating for low proficiency in fish capture until constrained by the short day lengths of late autumn [63]. This illustrates the ‘cost of having to learn’, where juveniles must invest more time and energy to achieve the same foraging success as adults. In addition to selecting less profitable foraging areas due to inexperience or an inability to assess resource abundance [14,65,66], the higher initial foraging time in juveniles may also reflect lower foraging skills and poorer food recognition [67,68], both of which can increase handling time [8]. Furthermore, competition with more experienced birds may lead to displacement to suboptimal areas or kleptoparasitism [8,19–21,69].

Over time, the juveniles roamed smaller areas and flew less, as originally predicted. However, we also hypothesized that juveniles would become more similar to adults over time as they would gain experience and learn. While we observed a temporal change in the differences between juveniles and adults, the increased similarity in spatial use, foraging effort and foraging efficiency appeared to be mainly driven by a change in adult behaviour. For example, we did not observe a decrease in cumulative distance travelled in juveniles, nor did they show a clear temporal pattern in the relative proportion of their time spent foraging. It remains unclear to what extent juveniles were sufficiently exposed to different landscape features during the 20 day period at the stopover sites to form spatial memories that would allow them to identify and return to profitable foraging areas within their range distribution area. Although spatial memory develops at a very young age in migratory birds, evidence from other species suggests there are neuronal (hippocampal) differences between juveniles and adults [70], indicating that the spatial memory of juveniles may be less developed than that of adults. Also, our study period may have been too short to show an improvement in juvenile food processing time, such as food handling time, that would allow them to exploit the foraging areas more efficiently. Repeating the study over a longer period, during subsequent stopovers or in wintering areas, may provide more robust evidence of improvement and trial-and-error learning.

The observation that adults roamed large areas upon arrival at the stopover site is consistent with the idea that initial exploration and identification of high-quality foraging areas can be considered adaptive for updating past environmental knowledge [71,72], as long as this does not involve high energy or time costs [73]. If so, adults may initially be willing to travel longer distances than naive juveniles while navigating to known profitable locations [74,75]. In addition, adults are often better than juveniles at detecting depleted foraging areas and deciding when to visit new areas [76]. As migrating adult lesser black-backed gulls tend to revisit stopover sites in different years [35], it is indeed highly plausible that most adults had some prior knowledge of the wider landscape when they reached their first stopover site. Given the lack of a clear temporal trend in the proportion of resting time (i.e. suggesting no significant time pressure), we suggest that the greater initial distances travelled and lower foraging time of the adults reflect a (directed) memory-guided exploration strategy [75], as opposed to ‘random exploration’. By contrast, when predicting that juveniles would explore more, we assumed their exploration would be more random due to their lack of prior experience. This idea of an adaptive, directed exploration in adults is further supported by the gradual decrease in their roaming behaviour, flight activity, distance travelled and the gradual increase in actual foraging with time spent at the site. Such optimization of spatial use and foraging behaviour after an initial exploration phase [77] reflects that adults perform an area-restricted search, switching from a broader search to a more focused search [78]. This is consistent with the results of a previous study of the same species, showing a gradual decrease in distances travelled and an increase in site fidelity with time [35], and of other adult seabirds that showed more specific exploration patterns over time with restricted foraging movements to good locations [9,10,59].

Although the results of this study support the idea that GPS tracking technologies can provide new insights into the study of spatial use and foraging strategies during early life, tracking data remain difficult to interpret without direct observations of important aspects of foraging behaviour, such as food intake. For example, in juveniles, a high proportion of foraging time may indicate lower foraging skills, such as slow and inefficient food intake. In contrast, for adults, it may reflect the effective location of suitable foraging sites, allowing more time to be spent on actual foraging. Also, GPS data rarely provide information about the social context, whereas large numbers of gulls usually gather in flocks when foraging [36,79]. Interactions with more experienced adults can provide valuable opportunities for juveniles to learn about the quality of foraging areas and further develop their foraging skills through observational learning [28,29,80,81]. The latter may be applicable to our study as juveniles migrated later than adults, so they could benefit from the experience of adults on arrival at their first stopover by following them to high-quality patches, thus reducing the need for extensive exploration [37,82]. In addition, as with most tracking studies, we lacked information on the quality of habitat patches. One possible GPS-based approach to solve these two issues is the use of heatmaps, generated from open-source databases of tracking data, which visualize hypothesized patches of high quality based on density. Finally, GPS tracking data collected at higher temporal resolution may provide more direct evidence of efficiency during spatial exploration and learning processes, for example, through the study of tortuosity [18,83]. Adults would typically show more direct flights to locations of interest, reflecting experience and spatial memory, whereas juveniles would initially show more tortuous flight paths that become more direct with learning over time [23,75].

## 5. Conclusion

In summary, our study reveals distinct foraging strategies between adult and juvenile lesser black-backed gulls during their first stopover. Although initial differences in spatial use, foraging effort and foraging efficiency between age classes tended to decrease over time, this pattern appeared to be driven mainly by changes in adult rather than juvenile behaviour. Overall, adults showed evidence of an exploration strategy, potentially based on previous knowledge, initially covering larger areas before optimizing spatial use and foraging behaviour over time. In contrast, juveniles showed no clear evidence of a gradual change in foraging-related behaviours, suggesting that spatial knowledge and foraging skills may still be too limited. While our results provide insights into the development of foraging behaviour in juveniles, further research is needed to elucidate learning processes and habitat quality, for example by combining observational studies of key foraging behaviours with higher resolution tracking data.

## Data Availability

The data and codes are available via Zenodo and Movebank. Intermediate data and scripts are provided through OSF [84]. The juvenile dataset analysed during the current study is available on Movebank, under study name LBBG_JUVENILE. The adult dataset analysed during the current study is available on Movebank, under study name DELTATRACK. Electronic supplementary material is available online [85].
